# Application of articaine in endoscopic endonasal dacryocystorhinostomy: a retrospective study

**DOI:** 10.3389/fmed.2024.1332793

**Published:** 2024-07-31

**Authors:** Shulin Liu, Li Shui, Zhaohui Liu, Qi Li

**Affiliations:** Chongqing Key Laboratory of Ophthalmology, Chongqing Eye Institute, Chongqing Branch (Municipality Division) of National Clinical Research Center for Ocular Diseases, The First Affiliated Hospital of Chongqing Medical University, Chongqing, China

**Keywords:** endoscopic endonasal dacryocystorhinostomy, EN-DCR, articaine, lidocaine, anesthesia effect

## Abstract

**Background:**

To investigate the comparative effects of local anesthesia using lidocaine with adrenaline vs. articaine with adrenaline in endoscopic endonasal dacryocystorhinostomy (EN-DCR).

**Methods:**

This retrospective study included a total of 180 patients. These patients were categorized into two groups: the lidocaine group, which received 2% lidocaine (1:100,000 adrenaline), and the articaine group, which received 4% articaine (1:100,000 adrenaline) for local anesthesia. The study compared anesthesia efficacy, intraoperative pain levels, intraoperative bleeding, as well as differences in heart rate and blood pressure between the two groups.

**Results:**

The articaine group demonstrated a significantly lower visual analog scale (VAS) pain score when compared to the lidocaine group, measuring at 4.4 ± 0.6 cm vs. 5.0 ± 1.0 cm, respectively (*P* < 0.0001). Additionally, the articaine group exhibited a higher anesthesia efficacy compared to the lidocaine group (89.0% vs. 76.6%, *p* = 0.0487). Notably, the articaine group experienced less nasal mucosal bleeding during the surgery in contrast to the lidocaine group (*p* = 0.004). However, there were no statistically significant differences in changes in blood pressure and heart rate between the two groups (*p* > 0.05).

**Conclusion:**

This study demonstrated that 4% articaine (1:100,000 adrenaline) has superior clinical effectiveness in comparison to 2% lidocaine (1:100,000 adrenaline) in EN-DCR.

## 1 Introduction

Endoscopic endonasal dacryocystorhinostomy (EN-DCR) combined with stent implantation stands as a highly effective surgical intervention for managing chronic dacryocystitis, demonstrating precise surgical outcomes and favorable postoperative results (1, 2). However, achieving successful surgical outcomes relies significantly on the clarity of the surgical field during the procedure and the patient’s cooperation (3). Most patients can undergo this surgery under local anesthesia, emphasizing the importance of selecting an appropriate local anesthetic to effectively manage intraoperative pain (4–6). In the past, the use of 2% lidocaine injection for anterior ethmoid nerve and infraorbital nerve block, as well as local infiltration anesthesia in the region of the uncinate process and its surrounding mucosa, has become a common practice in EN-DCR (3). However, we have observed that a notable portion of these patients experience suboptimal anesthetic effects during surgery, manifesting as pain, elevated heart rate, increased intraoperative blood loss, compromised surgical field visibility, and an overall negative patient experience.

Articaine is one of the most commonly used local anesthetics in the field of dentistry worldwide (7–9). Compared to other local anesthetics, it has excellent tissue penetration and high diffusion (10–13). Notably, its plasma protein binding rate can attain 95%, surpassing that of other agents (14).

To the best knowledge of the author, the anesthetic effectiveness of 4% articaine has not been studied in EN-DCR surgery. Hence, the aim of this study is to determine the effectiveness of 4% articaine (1:100,000 adrenaline) by comparing it to that of gold standard 2% lidocaine (1:100,000 adrenaline) in achieving adequate anesthesia in EN-DCR surgery.

## 2 Materials and methods

The patients’ records were collected in Department of Ophthalmology at the First Affiliated Hospital of Chongqing Medical University between February 2019 and February 2022. Only unilateral chronic dacryocystitis patients were included. Notably, the patients had no prior medical history of prevalent systemic conditions such as hypertension, diabetes, coronary heart disease, or autoimmune disorders. Additionally, none of the patients presented any nasal conditions such as deviated nasal septum or nasal polyps, and they had no record of allergic reactions to local anesthetics. This study received approval from the Ethics Board of the first affiliated hospital of Chongqing medical university. All patients provided signed informed consent for clinical research before undergoing any surgical procedures.

The surgical procedures and data recording for this study were conducted by authors Z. L, L. S, and S.L. The clinical operation steps were as follows (see Supplementary Video; Supplementary Figure 1).

### 2.1 Preparation and sterilization

Routine disinfection of the nasal and facial areas, followed by the placement of sterile surgical drapes to ensure aseptic conditions. Cotton swabs soaked in saline are placed in the inferior and common nasal meatus. Depending on the degree of wetness of the swabs during the procedure, they are replaced with new ones.

### 2.2 Anesthesia administration

#### 2.2.1 Surface anesthesia and mucosal constriction

Using nasal endoscopy, the affected side of the nasal cavity was filled with a cotton ball soaked in a blend of oxybuprocaine eye drops and adrenaline injection solution. This mixture served for surface anesthesia and mucosal constriction, maintained for a duration of 30 min.

#### 2.2.2 Lidocaine group anesthesia

In this group, patients underwent anterior ethmoid and infraorbital nerve block anesthesia using 1.7 mL of 2% lidocaine (1:100,000 adrenaline). Additionally, local infiltration anesthesia using 1.7 mL of 2% lidocaine (1:100,000 adrenaline) was administered around the uncinate process and surrounding mucosa.

#### 2.2.3 Articaine group anesthesia

In this group, patients underwent anterior ethmoid and infraorbital nerve block and local infiltration anesthesia using 1.7 mL of 4% articaine (1:100,000 adrenaline; Primacaine TM, France), respectively.

All injections were administered at a slow rate of approximately 1 mL/min to minimize trauma (15).

### 2.3 Surgical procedure

At least 3 min after anesthesia, the uncinate process and surrounding mucosa were lightly touched with a crescent knife tip, and the patient’s sensation was evaluated. We followed these steps to finish the procedure: (1) Create a posteriorly based mucosal flap to expose the lacrimal bone. (2) Raise a mucosal flap. (3) Remove the anterior ethmoidal bulla and frontal process of the maxilla to expose lacrimal sac. (4) The opening was gradually enlarged until a bony window was formed, exposing the inner wall of the lacrimal sac. (5) Marsupialize the lacrimal sac. (6) Trimming the nasal mucosal flap. (7) Inserting silastic stents.

### 2.4 Postoperative care and treatment

Postoperatively, patients received ice packs and appropriate medications for hemostasis and infection prevention.

During the aforementioned procedure, if the initial infiltration failed to achieve sufficient anesthesia and the patient was unable to tolerate the pain during surgery, this was verified by author Z.L. Subsequently, additional infiltration of the local anesthetic were administered as needed by author Z. L, following this protocol:

In the articaine group, in cases where the initial local anesthesia with articaine was ineffective, an additional infiltration of 0.5 mL of 4% articaine (1:100,000 adrenaline) was administered specifically to the uncinate process area.

In the lidocaine group, if the initial local anesthesia using lidocaine proved inadequate, an extra uncinate process infiltration of 0.5 mL 2% lidocaine (1:100,000 adrenaline) was administered.

### 2.5 Intraoperative and postoperative evaluations

The term “effective proportion (EP)” refers to the percentage of patients who achieved efficient anesthesia within 40 min and were able to cooperate with the surgery in a calm state. EP was calculated as the ratio of patients who experienced successful anesthesia to the overall patient count, and it is expressed as a percentage. During the surgery, the patients were asked to score the pain experienced during removal of the anterior ethmoidal bulla and frontal process of the maxilla on a 10 cm visual analog scale (VAS).

A noninvasive monitor (HEM-7071, Omron Corporatio, China) was utilized to record systolic and diastolic blood pressure (Sys. BP and Dia. BP, respectively) at 5-min intervals. Concurrently, heart rate (HR) was continuously recorded. Standard time points and time intervals were defined: (1) Baseline: beginning of monitoring; (2) LA: injection time; (3) LA + 5: 5 min after the injection; (4) End: completion of surgery.

During the surgical procedure, the amount of bleeding from cutting the uncinate process and its surrounding mucosa, as well as removing frontal process of maxilla and lacrimal bone, was collected using a suction device.

### 2.6 Statistical analysis

Data analysis was conducted using SPSS 20.0 statistical software. The chi-square test and independent sample t-test were used for statistical analysis. The level of significance adopted in our study was less than 0.05.

## 3 Results

In this study, a total of 180 patients were enrolled, comprising 76 males and 104 females, with ages ranging from 36 to 77. The mean age in the articaine group was 54.3 ± 6.3, and in the lidocaine group was 55.0 ± 7.0. No statistically significant difference was observed between the two groups in terms of age.

The effective proportion (EP) in the articaine group (*n* = 73) was 89.0%, while in the lidocaine group (*n* = 107) was 76.6%, the difference was statistically significant (*p* = 0.0487; see Table 1). The VAS scores in the articaine group and lidocaine group were (4.4 ± 0.6) cm and (5.0 ± 1.0) cm, respectively, with a statistically significant difference between the two groups (*P* < 0.0001). Nonetheless, there were no statistically significant differences observed in the heart rate, systolic blood pressure and diastolic pressure during the surgery between the two groups (see Table 2). On the other hand, a statistically significant difference was noted in the comparison of intraoperative bleeding volume between the two groups: 4.06 ± 0.94 mL in the articaine group and 4.43 ± 0.93 mL in the lidocaine group (*p* = 0.004).

**Table 1:** Comparison of anesthesia induction efficiency in the two groups of patients during surgery.

	Total cases	Successful induction (*n*)	Effective induction (*n*)	Failed induction (*n*)	Anesthesia induction efficiency (%)
Articaine group	73	65	9	8	89.0
Lidocoaine group	107	82	18	25	76.6

**Table 2:** Comparison of hemodynamic data between two groups.

	Timing	Articaine	Lidocoaine	*p*-value
HR1 (beats/min)	Baseline	67.5	68.5	0.4286
	LA4	68.1	68.6	0.6938
	∆5 (LA + 5)6-LA	2.38	2.64	0.3558
	∆ End7-LA	1.03	0.55	0.06
Sys. BP2 (mm Hg)	Baseline	129.9	128.7	0.6502
	LA	129.8	126.4	0.1364
	∆(LA + 5)-LA	1.19	1.73	0.1655
	∆ End-LA	3.34	3.53	0.8155
Dia. BP3 (mm Hg)	Baseline	72.3	74.8	0.0801
	LA	72.3	73.2	0.4517
	∆(LA + 5)-LA	1.58	0.92	0.1277
	∆ End-LA	1.64	1.35	0.5682

HR, heart rate; Sys. BP, systolic blood pressure; Dia. BP, diastolic blood pressure; LA, injection time; ∆, changes between different time points; (LA + 5), 5 min after the injection; End, completion of surgery.

Another parameter considered for comparing the efficacy of the two anesthetics was the need for additional anesthesia administered in each group. None of the patients in the articaine group required supplementary infiltration in the uncinate process area. Conversely, in the lidocaine group, the number of additional injections in the uncinate process area was 3.

Both groups of patients did not experience any adverse local anesthetic reactions such as injection site pain, rash, pruritus, drowsiness, nausea, and dizziness, palpitations, and sweat. The cardiovascular indicators were stable during the surgery.

## 4 Discussion

Surgery has become a crucial treatment option for chronic dacryocystitis. EN-DCR with stent implantation stands out as an effective approach, ensuring a clear surgical field and direct visualization through nasal endoscopy. In patients without underlying health conditions, this surgery can be conducted under local anesthesia, offering notable benefits including cost-effectiveness and rapid postoperative recovery. Nonetheless, if patients experience pain during local anesthesia, it may escalate bleeding and result in suboptimal exposure of the surgical field, leading to a decrease in surgery success rate. This discomfort can also trigger elevated blood pressure and heart rate, posing potential risks. Hence, in EN-DCR with stent implantation under local anesthesia, alleviating patient discomfort holds the potential to significantly enhance patient safety, and accelerate postoperative recovery.

Among various local anesthetics, lidocaine stands as the “gold standard.” However, articaine has recently gained notable recognition as an exceptional local anesthetic for dental procedures (16–18). In this study, the use of articaine led to a superior success rate in achieving anesthesia than lidocaine, especially during the removal of the frontal process of maxilla and lacrimal bone, markedly reducing the need for additional anesthetics during the procedure. It is important to emphasize that articaine is equally effective in inflamed tissues (19), for example, Narendrababu et al. (20) confirmed that articaine was more efficacious than lidocaine for anesthesia of teeth with irreversible pulpitis. This proves significantly advantageous in alleviating pain associated with incisions during EN-DCR, particularly in the chronic inflammatory stage of the lacrimal sac. One of the causes of anesthetic failure is that, due to inflammation, local anesthetic may show faster systemic absorption given the vasodilation promoted by the inflammatory process (21). The local pH will decrease in the inflamed tissue, which results in a decreased fraction of the neutral local anesthetic form and greater lipid solubility and membrane partitioning into the nervous membrane, where the voltage-gated sodium channel is located (19, 22, 23). The remaining effectiveness of articaine in inflamed tissues may be attributed to its distinct chemical structure, which differs from lidocaine by substituting the aromatic ring with a thiophene ring. This modification enhances articaine’s lipid solubility, contributing to its potency being approximately 1.5 times greater than that of lidocaine (19). Articaine also contains a methyl ester group, resulting in a higher protein binding rate (24). It is known that the higher the degree of binding of the local anesthetic molecule with the nerve membrane, the more prolonged is the anesthetic effect along with better pain control (25). Thus, even with the same injection technique, articaine may theoretically result in better anesthetic effect as compared to lidocaine (26). Moreover, the onset time of 4% articaine (1:100,000 adrenaline) is significantly less than that of 4% lidocaine (27). These demonstrate good compatibility with the human body, particularly in terms of achieving effective local infiltration anesthesia.

In our study, we observed a significant reduction in intraoperative bleeding volume within the articaine group when compared to the lidocaine group. Effective control of bleeding during surgery resulted in a clearer surgical field and decreased complexity for the surgeon. Articaine can also play a pivotal role in reducing patient anxiety, thereby enhancing the overall surgical experience (28). Since EN-DCR involves bone cutting and mucosal incisions, which could potentially cause psychological stress to patients, articaine is particularly well-suited for this procedure. Furthermore, alleviation of pain and patients anxiety, likely contributed to more stable hemodynamics. This observation is consistent with previous literature (12, 29).

In our study, the blood pressure and heart rate of patients in the articaine group remained stable during the surgery. This is attributed to the excellent safety profile of articaine. Articaine is considered one of the safest because its rapid metabolism into an inactive metabolite minimizes the potential for overdose and systemic toxicity, even after many injections (30). A previous study demonstrated that only 5% to 10% of articaine metabolism takes place in the liver, with the majority, 90% to 95%, occurring in the blood (31). Additionally, the plasma half-life of articaine is approximately 20 min, ensuring its safety during usage. In our study, we utilized Primacaine Adrenaline, maintaining a fixed dose of adrenaline during articaine local infiltration anesthesia. This approach minimized the variability associated with manually measured adrenaline doses and avoids the side effects linked to excessive adrenaline use. Consequently, this strategy leads to stable blood pressure and heart rate throughout the surgery and in the postoperative period.

This study has several limitations, primarily its relatively small sample size and retrospective design. The different concentrations of anesthetic agent (4% articaine vs. 2% lidocaine) could potentially act as a confounding factor. However, only 4% articaine is commercially available, and it’s worth noting that a literature review by Yapp et al. highlighted that articaine preparations with 4 and 2% concentrations demonstrated similar clinical efficacy (32). Therefore, using 2% lidocaine as the control group can also help elucidate the research question.

## 5 Conclusion

In conclusion, we observed that 4% articaine with 1:100,000 adrenaline for local anesthesia offered distinct advantages over using 2% lidocaine with 1:100,000 adrenaline in pain control in EN-DCR. Further multi-center prospective research is necessary to validate these findings.

## Data availability statement

The raw data supporting the conclusions of this article will be made available by the authors, without undue reservation.

## Ethics statement

The studies involving humans were approved by the Ethics Board of the First Affiliated Hospital of Chongqing Medical University. The studies were conducted in accordance with the local legislation and institutional requirements. The participants provided their written informed consent to participate in this study.

## Author contributions

SL: Data curation, Funding acquisition, Writing – original draft. LS: Formal analysis, Writing – review & editing. ZL: Investigation, Methodology, Writing – review & editing. QL: Conceptualization, Writing – review & editing.
