# Supplementary figures and images for: Application of articaine in endoscopic endonasal dacryocystorhinostomy: a retrospective study
Source: Front Med (Lausanne). 2024 Jul 31;11:1332793. doi: 10.3389/fmed.2024.1332793 (PMC11322971; doi:10.3389/fmed.2024.1332793)

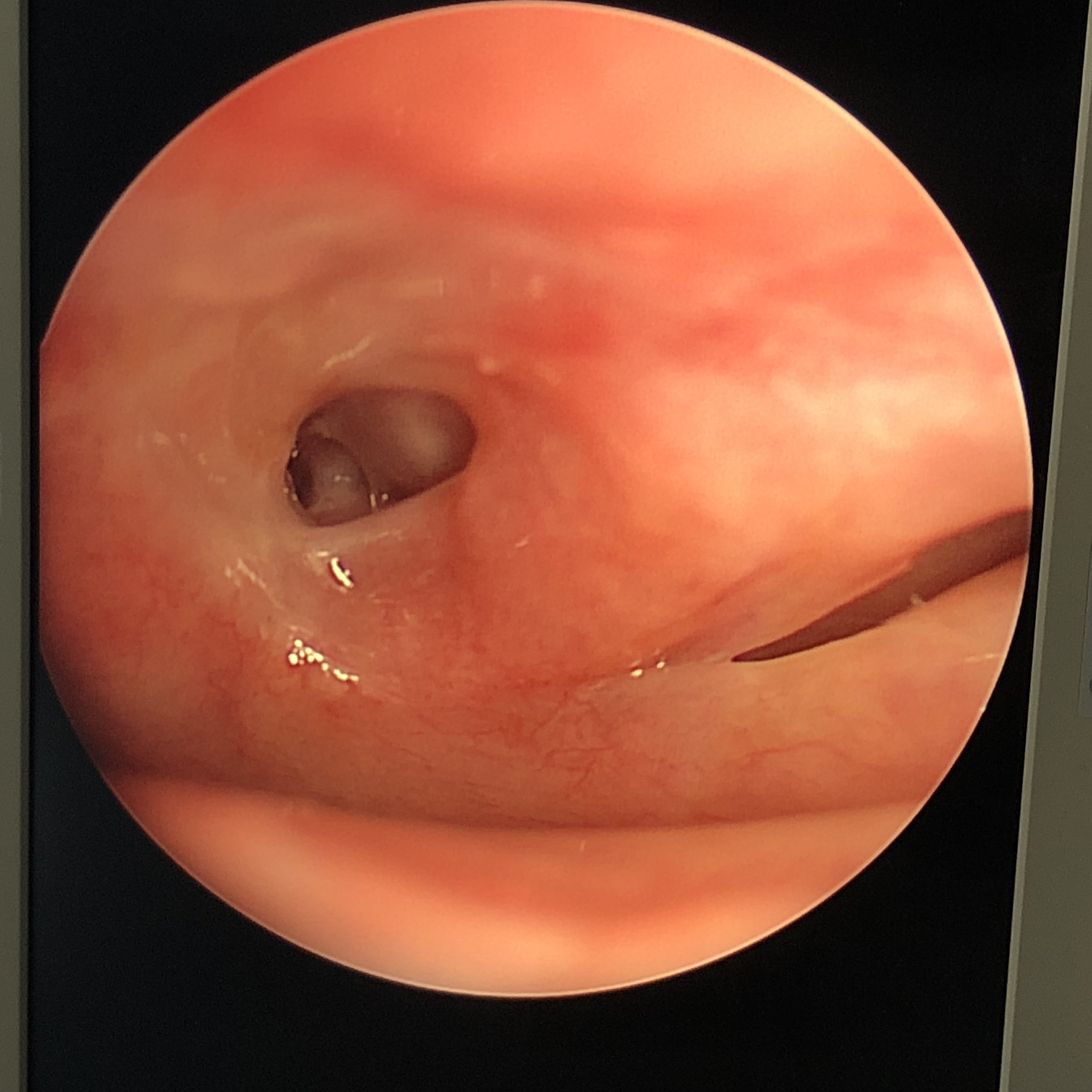

Supplement: Supplementary file 1 [file Image_1.JPEG]
